# Patients’ views and needs about systemic sclerosis and its management: a qualitative interview study

**DOI:** 10.1186/s12891-017-1603-4

**Published:** 2017-05-30

**Authors:** Luc Mouthon, Sophie Alami, Anne-Sophie Boisard, Benjamin Chaigne, Eric Hachulla, Serge Poiraudeau

**Affiliations:** 1 Pôle de Médecine Interne, Centre de référence pour les vascularites nécrosantes et la sclérodermie systémique, hôpital Cochin, Assistance Publique-Hôpitaux de Paris (AP-HP), Université Paris Descartes, Faculté de Médecine, Paris, France; 2 Interlis, Paris, France; 3 Service de Médecine Interne, Centre de référence pour la sclérodermie systémique, Hôpital Claude Huriez, Université Lille 2, Lille, France; 4 Faculté de Médecine, Service de Médecine Physique et Réadaptation, hôpital Cochin, AP-HP, Université Paris Descartes, Paris, France; 5 INSERM U1153, INSERM/CNRS Institut Fédératif de Recherche sur le Handicap, Paris, France; 6 Department of Internal Medicine, Cochin Hospital, 27, Rue du Faubourg Saint-Jacques, 75679 Paris Cedex 14, France

**Keywords:** Systemic sclerosis, Patients, Expectations, Healthcare strategies, Qualitative study

## Abstract

**Background:**

Systemic sclerosis (SSc) is a chronic connective-tissue disease responsible for reduced life expectancy, disability and a decreased quality of life. In order to optimize patients-physicians relationship and care strategy we aimed to survey views of patients on SSc and its management to reveal potential hurdles and improve health care strategies.

**Methods:**

A qualitative study combined semi-structured interviews, focus groups, and a direct observation of an information session was performed between November 2008 and January 2009.

**Results:**

Twenty-five patients with SSc were included. They encounter difficulties to have a clear representation of their disease. Physical, psychological, and social repercussions of SSc may lead to a psychological distress and different coping strategies, which widely differ among interviewed patients. Patients’ views on their therapeutic journey and the management of their disease highlighted strong expectations about patient-physician relationship. These expectations were numerous, complex and sometimes ambivalent. Patients expected physicians to be human and attentive but also involved in research in the field and to provide psychological and affective support to help them to accept the uncertainty of disease evolution and lack of curative treatment. They also expected more individualized management, improvements in diagnosis and follow-up organization, more efforts in education and information, comprehensive behaviors and support from working colleagues and relatives, and increased funding from the health care system.

**Conclusions:**

Our results suggest that SSc management could be optimized, particularly with more attention to the patient–practitioner relationship. Patient profiles should be more precisely defined in terms of coping strategies and treatment preferences to propose more individualized options.

## Background

Systemic sclerosis (SSc) is a connective-tissue disease characterized by skin and visceral excessive collagen deposition and by vascular hyper-reactivity and obliterative microvascular phenomena [1]. SSc management is predicated on identifying organ-specific disease manifestations and initiating adapted therapies [2]. Visceral involvement is responsible for reduced life expectancy [3–6] while the skin, tendon, joint, and vessel damages lead to impairment, disability, and a decreased quality of life (QoL) [7]. A primary goal of care is to reduce symptoms, disability and improve QoL. As for other chronic diseases, understanding views and needs of patients may lead to optimize patients-physicians relationship, share-decision making about treatment strategies, patients’ and caregivers’ education and therefore adherence to and efficacy of treatments [8–11].

Although patients and their physicians differ in their assessment of important health and symptom status in several chronic diseases, the views of patients concerning SSc and its management have seldom been studied [12–15].

The relevance of patients’ perspectives to the medical decision-making process and product development has been pointed out [16], and guidelines have been edited that broaden the traditional perspective of medicine by considering indicators of what “really matters” for patients as part of therapeutic assessment [17]. Qualitative research is probably the best way to understand patient needs and contexts and could improve therapeutic strategies and their assessment [18]. Indeed, the US Food and Drug Administration recently proposed guidelines for patient-reported outcomes that emphasize the need for semi-structured interviews of patients to ensure content validity of these instruments [19].

In the present study, we assess patients’ views about SSc. Our research questions were: (i) What are patients’ views of the disease, (ii) and of QoL in SSc? (iii) What are their evaluation and expectations regarding the care process?

## Methods

### Design

A qualitative study based on an inductive enquiry consistent with the grounded theory approach was adopted [20] and conducted in accordance with qualitative research guidelines [21, 22] was performed between November 2008 and January 2009. It combined 3 complementary strategies of data collection: semi-structured interviews, focus groups and a direct observation of an information and education session.

### Participants and sampling

Patients were selected from files of care providers belonging to 3 departments of internal medicine from 2 French University hospitals and with the help of a SSc-patient association, the Association des Sclérodermiques de France (ASF). The sample of people to be interviewed was selected on the basis of non-probability judgment sampling [23].

### Procedure

Face-to-face 1.5-h-long semi-structured interviews were conducted to assess interviewee’s personal experience. An interview protocol was developed from exploratory interviews of medical experts in the field. It explored patients’ views on SSc (its origins, mechanism, and evolution and their understanding of the illness); disease consequences, and the subsequent adjustments; description and evaluation of the patients’ therapeutic journey through the health system; their evaluation of SSc management (including the evaluation of patient–healthcare professional relationships, and treatments); and their expectations for improving health care management of SSc.

Three-hour focus group session was held after the interview session. It was designed to encourage discourse and comment on each participant’s experiences and views. It was led by a moderator who focused on issues that had emerged during the interviews. He combined projective exercises with directive questions and collage technique.

The direct observation technique was undertaken to provide insights into interactions between patients and medical staff in the specific setting of a 3-h information and education session. Specific attention was given to patients’ behavior and participation into the session.

#### Data collection

Interviews and focus group session were audio-recorded and fully transcribed with the interviewees’ agreement in order to complete the notes taken. Detailed field notes were taken during the observation session.

#### Analysis

The data set, the written transcripts, and the observation notes, were analyzed by 3 researchers (2 sociologists, one anthropologist) using the framework of thematic content analysis and categorized independently in accordance with the Consolidated criteria for reporting qualitative research (COREQ) [22]. Investigators read and re-read the transcripts and manually identified the key themes from the data. The first categorizing system was consequently modified, subcategories being added, as they emerged from the content analysis. Numerous free categories were developed, discussed and adjusted in an iterative and inductive process. This coding frame was used to systematically index the data. Finally, an anonymized and extensive document reporting the analysis was produced with numerous participant quotations to support the findings.

## Results

The study included 25 patients. Sixteen patients were individually interviewed and 9 patients participated to the focus group. Patients were interviewed at home (*n* = 9), in a convenient public location (*n* = 1), in a professional setting (*n* = 1) or at hospital (*n* = 7). Six patients and 1 relative of one patient took part in a 3-h information and education session allowing direct observation. The diversity of the interviewed patient sample and the focus group is shown in Table 1.

**Table 1:** Characteristics of the interviewed and the focus group patient population

Semi-interviewed patient population	
Feature	Composition of study population (*n* = 16)
Gender	- 12 females
Age	- 6 patients <50 years
	- 5 patients 50–65 years
	- 5 patients >65 years
Current work status	- 3 working
	- 4 non-working (1 patient on sick leave for 1 year and 3 disabled patients)
	- 9 retired patients
Place of living	- 13 urban
	- 3 rural
Disease manifestations^a^	- 4 patients with PAH
	- 4 patients with DU
	- 8 patients with other type of organ involvement
Focus group patient population	
Feature	Composition of study population (*n* = 9)
Gender	- 9 females
Age	- 4 patients <50 years
	- 4 patients 50–65 years
	- 1 patients >65 years
Current work status	- 4 working
	- 4 non –working (2 patients on sick leave and 2 disabled patients)
	- 1 retired
Household members	- 3 persons living alone
	- 4 cohabiting or married (2 with children)
	- 1 living with brother
	- 1 living with her student daughter and her daughter’s companion

*PAH* pulmonary arterial hypertension, *DU* digital ulcer

^a^declared by the patient interviewed

### Patients’ representation of SSc

**Fig. 1:**
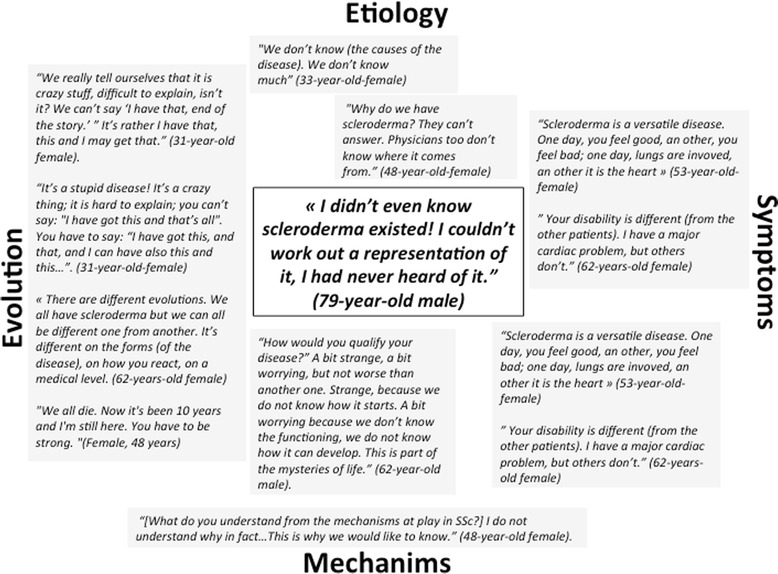
Patients’ representation of SSc

Patients’ representations of SSc were not univocal but rather polymorphic and had changed over time. This first finding highlighted a lack of understanding of the disease but it also showed that patients did not have certain knowledge of the disease. Their representation of SSc combined elements of symptoms, causes, diagnostic tools, results of complementary exams and treatments without any global representation of the disease. Patients considered SSc a new and mysterious illness, difficult to understand, difficult to decipher and difficult to explain. To assess their representation of the disease, they mainly relied on 4 attributes, which include potential mechanisms, disease etiology, disease evolution and somatic consequences (Fig. 1).

#### Representations of SSc mechanisms

Although disease mechanisms were not known for most patients, SSc was cited as a disease leading to an excessive and harmful production of collagen, antibodies, and fluid disorders.

#### Representation of SSc etiology

Interviewed patients were not able to assign an origin to the disease. As a consequence they had personal views on the cause of the disease: vaccines and adjuvants, emotional or psychological shocks (depression, morning, accident, stress, divorce); pregnancy; or a spiritual explanation, such as a divine test or a spiritual attack.

#### Representation of SSc evolution

When asked about SSc evolution, patients emphasized its erratic and unpredictable pattern. They pointed out the inter and intra-individual variability of the symptoms of the disease, the absence of identifiable logic to explain which organs are affected by the disease and either its incurability or its slow progression.

#### Representation of SSc symptoms

Symptoms played a pivotal role in the disease representation as they made the illness real by being “visible”. Patients combined different sources of information mixing personal experiences and medical discourses to build their own semiology of SSc. However, this representation was fragmented due to the multiplicity of the physical impact of the disease.

Patient descriptions of physical and physiological consequences of SSc differed from medical descriptions. They identified 2 categories of symptoms. They mentioned localized manifestations in opposition to functional symptoms. In the former category, they included lungs, heart, kidney, skin and more particularly face and hands, musculoskeletal system, digestive system, genital system and/or cognitive functions involvement. Raynaud’s phenomenon was also part of this category due to its localization on hand and feet. In the latter category, patients complained about general manifestations of SSc with functional disability including pain, fatigue, dryness and limitation in joint mobility.

### Patients’ views of their quality of life and coping strategies to face SSc (Fig. 2)

#### Impact of SSc on daily life

The consequences of SSc on daily living activities varied with time and disease state. Patient’s descriptions of living with the disease ranged from bothersome to impossible to live with. Described repercussions concerned every domain of daily life, including motility, nutrition, psychology (fears, anxiety, mood and sleep disturbances, biographic rupture, identity crisis), occupational activities, social life, and incomes (decreased income and increased costs directly or indirectly related to the disease). SSc affects, in functional terms, household chores, professional activities, leisure activities (sports, shopping, cultural and religious activities), body care and dressing, parenting, sexuality, mobility, and medical care.

**Fig. 2:**
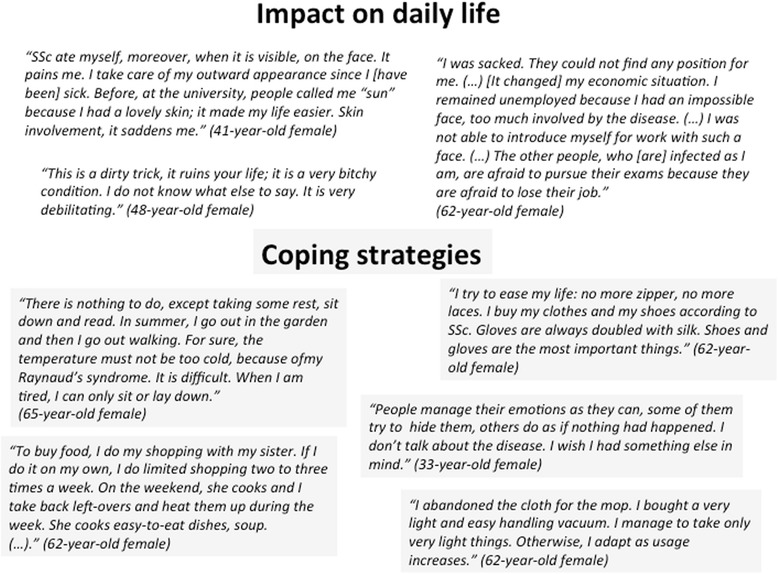
Patients’ views of their quality of life and coping strategies to face SSc

#### Coping strategies

Because of above-mentioned limitations patients reported a renouncement of a “previous life” and the need for constant efforts to adapt themselves to new constraints imposed by their health-related situation. Confronted with these major changes, patients adopted different coping strategies.

On one hand, some patients reported different strategies including environment adaptation, time adaptation and/or social adaptation. They tried to adapt their material environment to protect their independence, avoiding objects or products that they can no longer use and adopting more convenient ones. Adaptation in organizing time was also reported. Patients had to slow down their life rhythm, adapt or edit their projects and sometimes abandon them.

Relying on social and/or family support was another coping strategy. However the relationship between the patient and the helper could lead to tension, mutual incomprehension or paradoxical injunction. These troublesome occurred when the support was considered insufficient by patients or when patients’ limitations were denied or undermined by the helper.

On the other hand, some patients avoid coping strategies, with an unwillingness to change their lifestyle or refusing to be affected by the disease. Some of them even hide their disease from siblings.

### Patients’ evaluation of the therapeutic journey and expectations on the care process

Three main themes were highlighted during the interview process: the therapeutic journey, views on management, and expectations for management.

#### Therapeutic journey (Fig. 3)

##### Deciding to consult

Patients’ decisions to seek consultation depended on the progression of disease (insidious vs. rapidly evolving) and age at symptom onset (youngest being less likely to consult quickly). The choice of health care professional was related to geographical location, relational proximity (general practitioners), or specialist health care professional (the choice depending on the main symptoms).

**Fig. 3:**
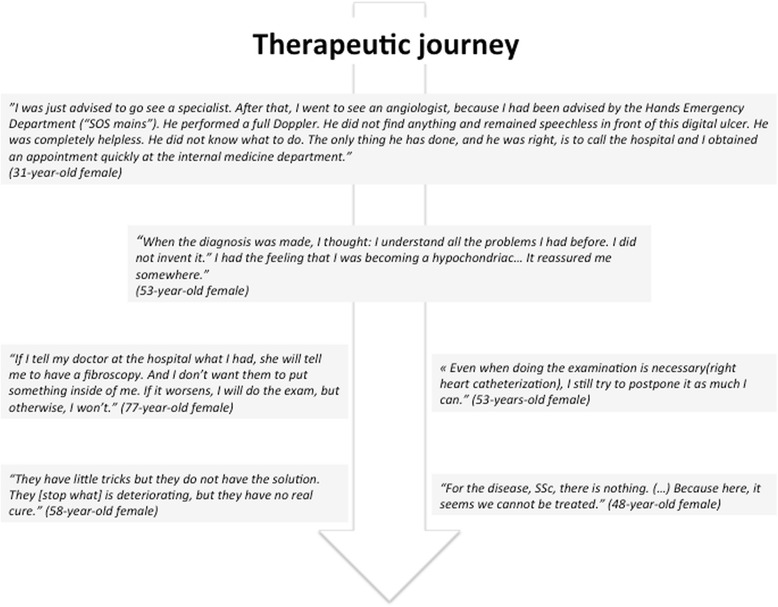
Patients’ evaluation of the therapeutic journey

##### Diagnosis

The time elapsed between the first symptoms and diagnosis varied widely, depending on the severity of the disease or the medical journey. The length of the patient journey before SSc diagnosis was made was attributed to the variety of health care professionals consulted, the progressive process of determination of their symptoms, and sometimes the difficulty in getting to an expertise center. Patients considered diagnosis as the pivotal event of the therapeutic journey. Blood exams and search for antibodies were identified as key events in the diagnosis process of SSc. The way the diagnosis was delivered and the words used to announce the diagnosis were important and induced various reactions: emotional reactions (anger, fear, anxiety); cognitive reactions (minimizing the announcement or denial; ambivalent search for information on causes, underlying mechanisms, implications, treatments, managements, prognosis); the use of the Internet for more information; and organizational reactions (anticipating evolution, and projection into the future). The diagnosis announcement was also a source of reassurance and recognition of their complaints.

##### Complementary exams

Patients recognized the usefulness of complementary examinations but emphasized that were source of anxiety since it could lead to the discovery of additional pathologies related to SSc. Some patients reported having hidden their symptoms to avoid or postpone fibroscopy. Right heart catheterism and fibroscopy were the two main complementary examination discussed as they generate pain, anxiety and constraints.

#### Views of management

To patients, SSc management relies on the relationship they share with their physicians, the information they receive about the disease and the treatments.

##### Relationships between patients and physicians

Confidence in the physician was considered as the pivotal element of the relationship. The feeling of confidence in physicians appeared to be determined by the combination of medical skills, interpersonal skills, accessibility, and the ability to individualize the patient–physician relationship. Overall frankness, ability to listen and technical skills were expectations, when fulfilled that brought satisfaction to patients. Oppositely, factors of dissatisfaction included, uncertain or diverging advice, absence of prescription of an active treatment, and lack of regularity of follow-up (missed appointments, absence of administrative support, etc.) and finally a lack of humanity (lack of tact, availability, interest in patients’ pain or therapeutic journey).

##### Information about the disease

The information received varied from satisfactory to insufficient. When patients expressed dissatisfaction about information, the limited level of knowledge or understanding of the disease was emphasized. Patients considered information given by care providers as brief and pragmatic. They recalled management, treatments, prognosis, and mechanisms of the disease. Concerning treatments, patients’ views of counseling were ambivalent. Although wanted, patients did not always ask for counseling and were not sure about what counseling should focus on.

Apart from medical advice, Internet, associations of patients, and educational programs were reported to be other available sources of information. The assessment of these sources of information was ambivalent; although researching information provided reassurance, it also caused anxiety and uncertainty.

##### Treatments

Patients distinguished pharmacological treatments, non-pharmacological treatments, and complementary medicine. Pharmacological treatments were categorized as long-term treatments for SSc and specific treatments for specific impairments. Patients’ assessment of these treatments was mainly negative, focusing on the absence of curative effects and the unpredictable character of symptomatic effects of these drugs. They emphasized on the constraints of taking these treatments; the number of treatments was considered important and associated with side effects. Attitudes regarding prescriptions were articulated along an axis of “passive behaviors” to “strong implication,” with practices including self-medication, self-management, and abandonment.

#### Expectations for management (Fig. 4)

Expectations for management were numerous, demanding, evolving, and sometimes ambivalent. Patients expected a holistic approach of their situation taking into account the physical damages and the impacted QoL. Every impacted dimensions of the disease including functional, social, psychological, professional and financial aspects were expected to be addressed, directly or with the help of other professionals. Pain and fatigue were mentioned as specific issues insufficiently addressed by physicians.

**Fig. 4:**
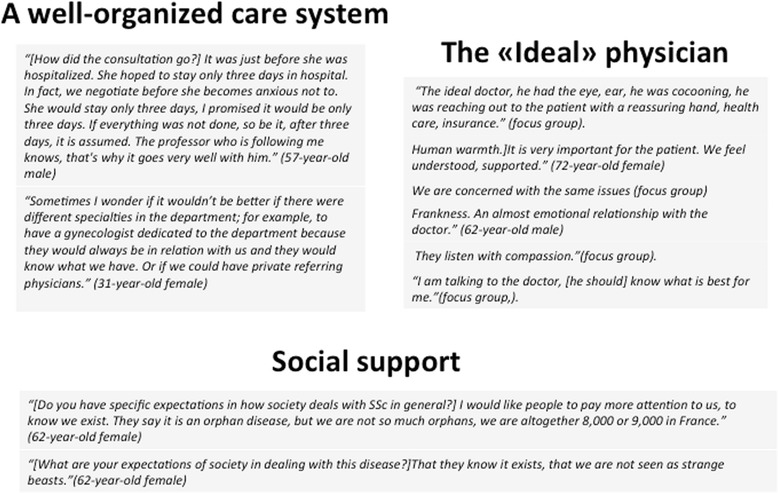
Patients’ expectations on the care process

Expectations regarding the relationship highlighted a need for individualization including availability, listening, and capacity of adaptation to patients no matter of social differences.

The ideal physician was defined by humanity and involvement in research, medical skills being a major and necessary condition but not at all sufficient. He was described as “The Savior”, an omnipotent figure that would be able to cure the disease.

Patients suggested a number of complementary axes to improve the management of their disease in order to obtain a well-organized care system: (i) improvement of hospital care, during hospitalization or in outpatient clinics (regularity of complementary examinations, quality of follow-up, improvement of hospitalization conditions through individualization of care), (ii) improvement of coordination between physicians:, and (iii) improvement of information on the specificities of SSc provided to health care professionals and relatives.

## Discussion

Our study provides new insights about the way patients consider SSc and its management. The qualitative design of the study provided extensive information on patients’ views about their symptoms, the impacts of SSc on daily life, and their expectations. These data could help in prioritizing areas of improvement in medical care.

Consistent with previous qualitative studies on SSc, our work confirms patients’ experience of the disease, mainly by its physical burden, social restriction and the disease navigating uncertainty [12, 24–27]. Interestingly, we collected new information regarding patients’ representation of SSc. Noteworthy we were able to identify coping strategies and patients expectations regarding the disease therapeutic journey and its overall management.

Although presented in a different manner, the main themes highlighted by our work were indeed consistent with previous qualitative studies on SSc. Thus, Joachim et al. identified five themes, which were of interest to SSc patients: physical manifestations, disclosure/non-disclosure to others, living, being normal and facing the future [13]. In addition, in a study involving 63 patients from four European countries, 19 concepts were shared among SSc patients from the four countries which included among others impaired hand function, household activities, paid work, drugs, climate and coldness, support from others and experiences with healthcare institutions, non-pharmacological treatment, social security and benefits [12]. Moreover, it has recently been reported that SSc patients experience difficulties regarding emotional, physical and social aspects and that individual abilities to cope with the disease were much more improved among patients who have a sustained social support [14]. Interestingly, none of the published studies identified the patient–physician relationship as of strategic importance in the care of SSc patients.

Our results indicated that patients strived to assign meaning to the changes they undergo and to devise a clear representation of their illness. Authors suggest that patients construct their own common-sense model of their medical situation and that illness representations determine coping responses, which influence health outcomes [28–32]. Empirical studies indicated that illness representations influence coping and outcomes in many diseases, such as chronic fatigue syndrome [33], neuroepilepsy [34], Addison’s disease [35], psoriasis [36], multiple sclerosis [37], or hypertension [38]. As expected, coping strategies differed among SSc patients. Coping strategies are rarely evaluated in SSc and their roles in the repercussion of the disease on QoL are largely unknown. Arat and colleagues have evaluated the contribution of illness perceptions and coping strategies on physical and mental health of SSc patients and concluded that “illness representations are more significant contributors to physical and mental health than classical disease characteristics” and that they should be taken into account [30]. Recording coping strategies of patients with SSc could be of interest to determine if certain ways of coping are beneficial or deleterious regarding the repercussions of the disease. If it is the case, behavioral therapy programs could be of interest for SSc patients. Altogether, these data argue for developing education programs in SSc. They should include an evaluation of patients’ illness representations in order to adjust the manner in which patients organize their lay beliefs about their illness and construct their coping strategy. These programs should therefore be personalized according to the patient’s interpretation of his own clinical and personal situation.

Our results suggest potential facilitators to improve SSc management. Patients expected a shift in the management of SSc from a technical viewpoint, to a more global approach. The stake is to promote SSc management strategies that will not be limited to physical symptoms but will take into consideration the impact of SSc on symbolic, temporal, relational, psychological, emotional, material, and physical dimensions. Patients emphasized the strategic importance of the patient–physician relationship in their satisfaction with SSc management, the necessary flexibility of this relationship, and the risk of the “routinization” of management in chronic clinical situations being an obstacle to the adaptation of this management to the specificities of the patient’s profile. The “ideal” patient-physician relationship is characterized by its flexibility; satisfaction cannot be considered a simple accumulation of factors. Physicians adjusting their behavior and practice to the patient seem to be pivotal in satisfaction. Practitioners should give satisfaction in consumerism, as well as technical, social and moral skills, and patients expect that physicians undergo a perpetual adaptation to the changing states and profiles of their patients. Dealing more accurately with some patient complaints such as fatigue and pain, not considered pivotal symptoms by physicians, may also be a way to improve SSc management. This issue raises the question of the absence of consensus on what is important between patients and physicians and should lead to more attentive and less “routine” attitudes during visits.

Along the same line, qualitative studies on SSc are important as they emphasize important issues for patients [12, 24–27]. Unfortunately, despite their importance, personal factors are not covered enough by patient-reported outcome measures [25]. In our work, patients explicitly expressed their will to be taken care in a well-organized care system with an “ideal” physician and their need for social support to help them coping with the disease. Altogether, previous qualitative studies on SSc and our work advocate a multidisciplinary approach to take care of patients with SSc. Such approach should therefore include biological, physical, psychological and importantly social assessment and management. However, our work clearly argues for a dedicated consultation allowing assessment of patients’ representation of the disease in order to answer patients questions and clarify their uncertainty about SSc.

Our study has several strengths. It combines 3 different data collection techniques addressing a large breadth of topics and providing different types of data that broaden the understanding of the social situation at stake and allow expectations to be understood in terms of their social context of emergence. Individual behaviors, personal feelings and interpretations, social interactions and material backgrounds were specifically examined throughout the patient’s therapeutic journey through the health system, thus allowing for a comprehensive analysis of patients’ expectations. The focus group approach allowed for delving deeper into patient beliefs, perceptions and knowledge of SSc. It also let people discuss SSc management, confront their expectations and express their perceptions of ideal care and physician–patient relationships. Direct observation allowed identifying, how physician succeeded or failed to meet patients ‘expectations and the importance of group dynamics on participants.

Our work has some limitations. It was performed in France. How the health system is organized, particularly with the specific French policy on infrequent diseases, which has led to the creation of reference centers, and the cultural context may have affected the views and expectations. Transcultural qualitative studies are needed to address this same question. Secondly, patients were selected on the basis of non-probability judgment sampling without taking the length of the disease and the disease subtype into consideration. These two factors are likely to impact patients views and expectations about SSc. Lastly it is impossible to differentiate between patients’ declared expectations and real ones. Indeed for some patients, declared expectations were possibly more a need for a reassurance than a need for information.

## Conclusions

In conclusion, our work underlines the views that patients have about SSc, which may limit the management of SSc more than the material constraints or the social dynamics of the patient–care provider relationship. More attention should be paid to patient views to increase their satisfaction with care and probably treatment adherence. Our results suggest several potential improvements to maximize management of SSc: more attention and time devoted to the patient–practitioner relationship and environmental factors. Education and information should be more formalized and proposed early in the course of the disease, including clinical manifestations related to lung involvement and complimentary exams performed to identify them. Patient profiles should be more precisely defined in terms of illness representations, coping strategies and treatment preferences to propose more personalized options.

## Abbreviations

ASF: Association des Sclérodermiques de France
QoL: Quality of life
SSc: Systemic sclerosis
